# Supplementary material for: Immunocytochemical Analysis of the Wall Ingrowths in the Digestive Gland Transfer Cells in Aldrovanda vesiculosa L. (Droseraceae)
Source: Cells. 2022 Jul 16;11(14):2218. doi: 10.3390/cells11142218 (PMC9322817; doi:10.3390/cells11142218)
Supplement: Supplementary file 1 [file cells-11-02218-s001.zip › cells-1774065-supplementary.pdf]

Figure S1

Figure S1. Control reactions of the immunolabeling of cell wall components. (**A-B**) Digestive gland; secretory cell (star), stalk cell (Sc), basal cell (Bc), bar 10 μm. (**C**) Immunogold labeling of cell wall components in gland cell; cell wall (cw), cell wall ingrowths (wi), bar 300 nm.

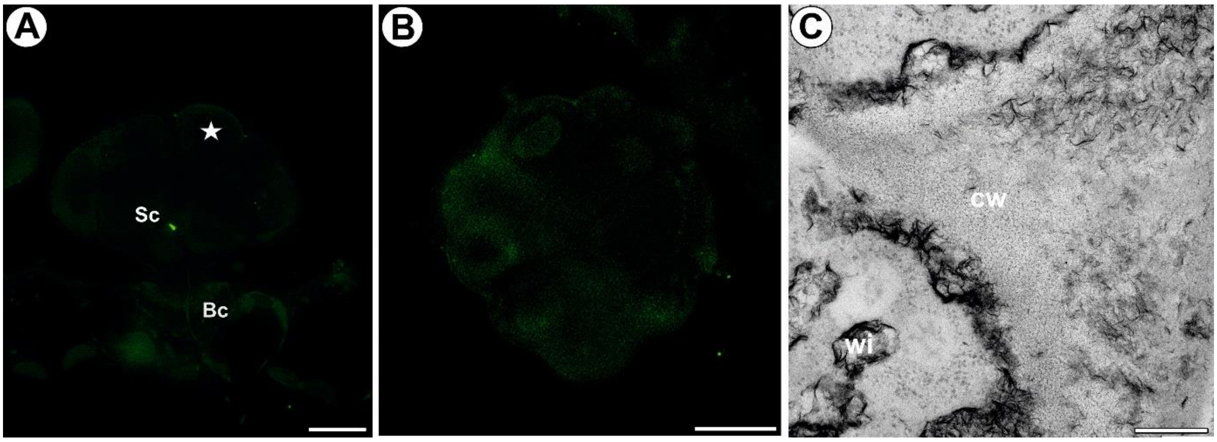

Table S1

Table S1. Statistical analysis of immunofluorescence labeling. Quantification of immunofluorescence labeling for the head cell wall. Mean value of AGP fluorescence intensity (MFI) for the negative control reaction (CTRL) and for labeled AGP (JIM14, JIM8, and JIM13 epitopes), pectins (JIM5, JIM7, LM5, LM6, and LM19 epitopes), hemi-celluloses (LM 15 and LM25 epitopes), and heteromannans (LM21 and LM22 epitopes) in 3 glands from 3 different traps (n = 3). Significance shown in red.

	one-way ANOVA effect SS	effect Df	effect MS	error SS	df	error MS	F	p-level
**Head cell wall MFI**	10113062	12	84275.2	1690478	26	65018.40	12.96179	0.000000
**Epidermal cell wall MFI**	1498320	12	124860.0	408621.8	26	15716.22	7.944656	0.000006
**Stalk cell wall MFI**	3255209	12	271267.4	320072.6	26	12310.48	22.03548	0.000000
**Ingrowths of basal cell wall MFI**	13856755	12	1154730	430942.3	26	16574.70	69.66819	0.000000

Tukey; Head cell wall MFI p < 0.05000

Epitope	JIM5 M=477.29	JIM7 M=430.40	JIM8 M=1345.4	JIM13 M=1660.5	JIM14 M=1658.5	LM5 M=798.36	LM6 M=399.07	LM15 M=1400.0	LM19 M=741.38	LM21 M=362.43	LM22 M=369.99	LM25 M=1168.7
**CTRL** M=209.59	0.981368	0.996272	0.000699	0.000156	0.000156	0.248190	0.999103	0.000421	0.383054	0.999895	0.999828	0.005107
**JIM5**		1.000000	0.014637	0.000448	0.000456	0.932161	1.000000	0.007807	0.983249	0.999995	0.999998	0.097681
**JIM7**			0.008540	0.000307	0.000311	0.848242	1.000000	0.004521	0.944985	1.000000	1.000000	0.060681
**JIM8**				0.939978	0.942403	0.343503	0.005926	1.000000	0.218572	0.003870	0.004224	0.999545
**JIM13**					1.000000	0.015664	0.000253	0.984984	0.008142	0.000212	0.000219	0.497058
**JIM14**						0.016018	0.000255	0.985858	0.008331	0.000213	0.000221	0.502910
**LM5**							0.771425	0.222919	1.000000	0.666745	0.689244	0.842948
**LM6**								0.003136	0.899096	1.000000	1.000000	0.043580
**LM15**									0.133900	0.002047	0.002247	0.994381
**LM19**										0.822941	0.840526	0.692389
**LM21**											1.000000	0.029265
**LM22**												0.031800

Tukey; Epidermal cell wall MFI p < 0.05000

Epitope	JIM5<br>M=563.88	JIM7<br>M=533.03	JIM8<br>M=383.59	JIM13<br>M=755.27	JIM14<br>M=341.34	LM5<br>M=771.80	LM6<br>M=431.75	LM15<br>M=603.72	LM19<br>M=546.03	LM21<br>M=265.24	LM22<br>M=286.02	LM25<br>M=798.59
**CTRL**<br>M=169.69	0.030745	0.060217	0.669313	0.000419	0.886726	0.000319	0.379767	0.012361	0.045557	0.998858	0.993179	0.000230
**JIM5**		1.000000	0.850989	0.797497	0.616079	0.705295	0.980801	1.000000	1.000000	0.212283	0.299525	0.540423
**JIM7**			0.952874	0.617943	0.795945	0.515432	0.998019	0.999945	1.000000	0.349457	0.465502	0.361063
**JIM8**				0.050388	1.000000	0.035122	0.999999	0.631049	0.918670	0.992106	0.998609	0.019208
**JIM13**					0.019684	1.000000	0.134674	0.948151	0.697410	0.003294	0.005389	1.000000
**JIM14**						0.013447	0.999328	0.378033	0.724228	0.999881	0.999996	0.007155
**LM5**							0.097441	0.899877	0.596002	0.002240	0.003644	1.000000
**LM6**								0.885502	0.994149	0.905346	0.960409	0.055900
**LM15**									0.999994	0.100542	0.150328	0.779183
**LM19**										0.285983	0.390956	0.432980
**LM21**											1.000000	0.001202
**LM22**												0.001934

Tukey; Stalk cell wall MFI p < 0.05000

Epitope	JIM5<br>M=585.11	JIM7<br>M=497.40	JIM8<br>M=796.68	JIM13<br>M=983.14	JIM14<br>M=949.16	LM5<br>M=731.33	LM6<br>M=584.37	LM15<br>M=1168.9	LM19<br>M=544.50	LM21<br>M=367.61	LM22<br>M=349.33	LM25<br>M=1122.2
**CTRL**<br>M=239.96	0.033766	0.242416	0.000230	0.000149	0.000149	0.000747	0.034397	0.000149	0.089799	0.963217	0.988716	0.000149
**JIM5**		0.998391	0.513736	0.008564	0.020886	0.909731	1.000000	0.000183	1.000000	0.473189	0.356621	0.000299
**JIM7**			0.101198	0.000858	0.002033	0.367651	0.998513	0.000151	0.999997	0.958646	0.902602	0.000158
**JIM8**				0.688790	0.884286	0.999913	0.508627	0.016893	0.267491	0.003730	0.002297	0.054791
**JIM13**					1.000000	0.269286	0.008397	0.693489	0.002890	0.000161	0.000156	0.934242
**JIM14**						0.470988	0.020492	0.457896	0.007162	0.000186	0.000169	0.775851
**LM5**							0.906924	0.002972	0.686322	0.021062	0.013073	0.010355
**LM6**								0.000182	1.000000	0.478188	0.361010	0.000295
**LM15**									0.000158	0.000149	0.000149	0.999998
**LM19**										0.751755	0.628582	0.000191
**LM21**											1.000000	0.000149
**LM22**												0.000149

Tukey; Ingrowths of basal cell wall MFI p < 0.05000

Epitope	JIM5<br>M=415.51	JIM7<br>M=485.41	JIM8<br>M=696.51	JIM13<br>M=1288.3	JIM14<br>M=1453.7	LM5<br>M=622.80	LM6<br>M=769.70	LM15<br>M=2232.2	LM19<br>M=381.08	LM21<br>M=320.31	LM22<br>M=334.31	LM25<br>M=1624.5
**CTRL**<br>M=229.17	0.845765	0.450721	0.007552	0.000149	0.000149	0.039130	0.001411	0.000149	0.956071	0.999439	0.997804	0.000149
**JIM5**		0.999963	0.320049	0.000149	0.000149	0.740566	0.088362	0.000149	1.000000	0.999144	0.999823	0.000149
**JIM7**			0.719158	0.000150	0.000149	0.978875	0.304416	0.000149	0.997955	0.923763	0.957672	0.000149
**JIM8**				0.000494	0.000152	0.999935	0.999940	0.000149	0.182593	0.056513	0.075264	0.000149
**JIM13**					0.922848	0.000197	0.002324	0.000149	0.000149	0.000149	0.000149	0.125094
**JIM14**						0.000149	0.000177	0.000151	0.000149	0.000149	0.000149	0.906108
**LM5**							0.965322	0.000149	0.536283	0.227764	0.285362	0.000149
**LM6**								0.000149	0.043537	0.011373	0.015624	0.000149
**LM15**									0.000149	0.000149	0.000149	0.000379
**LM19**										0.999992	1.000000	0.000149
**LM21**											1.000000	0.000149
**LM22**												0.000149
